# Selective ablation of the ligament of Marshall reduces ischemia and reperfusion-induced ventricular arrhythmias

**DOI:** 10.1371/journal.pone.0203083

**Published:** 2018-08-28

**Authors:** Ruisong Ma, Zhiliang Qin, Xiaomei Yu, Shan Liu, Weiyi Qu, Huihui Hu, Da Luo, Zhibing Lu, Hong Jiang

**Affiliations:** 1 The Department of Cardiology, Renmin Hospital of Wuhan University, Wuhan, PR China; 2 Cardiovascular Research Institute, Wuhan University, Wuhan, PR China; 3 Hubei Key Laboratory of Cardiology, Wuhan, PR China; 4 Department of Cardiology, Central Hospital of Enshi Tujia and Miao Autonomous Prefecture, Enshi City, Hubei, China; Emory University, UNITED STATES

## Abstract

Cardiac sympathetic tone overdrive is a key mechanism of arrhythmia. Cardiac sympathetic nerves denervation, such as LSG ablation or renal sympathetic denervation, suppressed both the prevalence of VAs and the incidence of SCD. Accumulating evidence demonstrates the ligament of Marshall (LOM) is a key component of the sympathetic conduit between the left stellate ganglion (LSG) and the ventricles. The present study aimed to investigate the roles of the distal segment of LOM (LOM_LSPV_) denervation in ischemia and reperfusion (IR)-induced VAs, and compared that LSG denervation. Thirty-three canines were randomly divided into group 1 (IR group, n = 11), group 2 (LOM_LSPV_ Denervation + IR, n = 9), and group 3 (LSG Denervation + IR, n = 13). Hematoxylin-Eosin (HE) and Immunohistochemistry staining revealed that LOM_LSPV_ contained bundles of sympathetic but not parasympathetic nerves. IR increased the cardiac sympathetic tone [serum concentrations of noradrenaline (NE) and epinephrine (E)] and induced the prevalence of VAs [ventricular premature beat (VPB), salvo of VPB, ventricular tachycardia (VT), VT duration (VTD) and ventricular fibrillation (VF)]. Both LOM_LSPV_ denervation and LSG denervation could reduce the cardiac sympathetic tone in Baseline (BS) [heart rate variability (HRV)]. Compared with group 1, LOM_LSPV_ denervation and LSG denervation similarly reduced sympathetic tone [NE (1.39±0.068 ng/ml in group 2, 1.29±0.081 ng/ml in group 3 vs 2.32±0.17 ng/ml in group 1, P<0.05) and E (114.64±9.22 pg/ml in group 2, 112.60±9.69 pg/ml in group 3 vs 166.18±15.78 pg/ml in group 1, P<0.05),] and VAs [VT (0±3.00 in group 2, 0±1.75 in group 3 vs 8.00±11.00 in group 1, P<0.05) and VTD (0 ± 4 s in group 2, 0±0.88s in group 3 vs 10.0 ± 22.00s in group 1, P<0.05)] after 2h reperfusion. These findings indicated LOM_LSPV_ denervation reduced the prevalence of VT by suppressing SNS activity. These effects are comparable to those of LSG denervation. In myocardial IR, the anti-arrhythmic effects of LOM_LSPV_ Denervation may be related to the inhibition of the expression of NE and E.

## Introduction

Reperfusion therapies are the most effective therapies for myocardial infarct. However, in addition to its beneficial effects, reperfusion also causes kinds of injuries, including ventricular arrhythmias (VAs) or even lethal ones. As the annual incidence of emergency percutaneous coronary intervention and heart transplantation grows larger and larger, methods aimed at preventing reperfusion-induced VAs should be given more attention.

Autonomic nervous imbalance manifesting with over-activated sympathetic drive and withdrawn vagal tone was markedly associated with adverse cardiac events in a model of myocardial ischemia [1]. The activation of the cardiac sympathetic nervous system (SNS), especially the left stellate ganglion (LSG), worsens the prevalence of VAs and sudden cardiac death (SCD) [2]. Conversely, cardiac SNS denervation, such as LSG ablation or renal sympathetic denervation, suppressed both the prevalence of VAs and the incidence of SCD [3–5].

The ligament of Marshall (LOM), originates from the coronary sinus (CS) and ends in the pericardium near the left superior pulmonary vein (LSPV) and is subdivided into a proximal portion (LOM_CS_), a middle portion and a distal portion (LOM_LSPV_). LOM_LSPV_ had been demonstrated richly innervated by sympathetic nerves [6]. Amounts of studies proved the key effect on LOM on the electrophysiology of atrium and LOM ablation should be an efficient approach to treat atrial arrhythmia [7, 8]. However, the effect of LOM on ventricular electrophysiology is rarely reported. Recently, our group found that LOM is a key component of the sympathetic conduit between the LSG and the ventricles. LOM_LSPV_ Denervation inhibited increases in blood pressure and the prevalence of ventricular tachycardia (VT) and ventricular fibrillation (VF) in response to stimulation with LSG in a Cesium-induced long QT model [9]. The present study was designed to investigate the effects of LOM_LSPV_ denervation on IR-induced VAs and compared these effects with those of LSG denervation.

## Methods

### Animal preparation and experimental design

**Fig 1:**
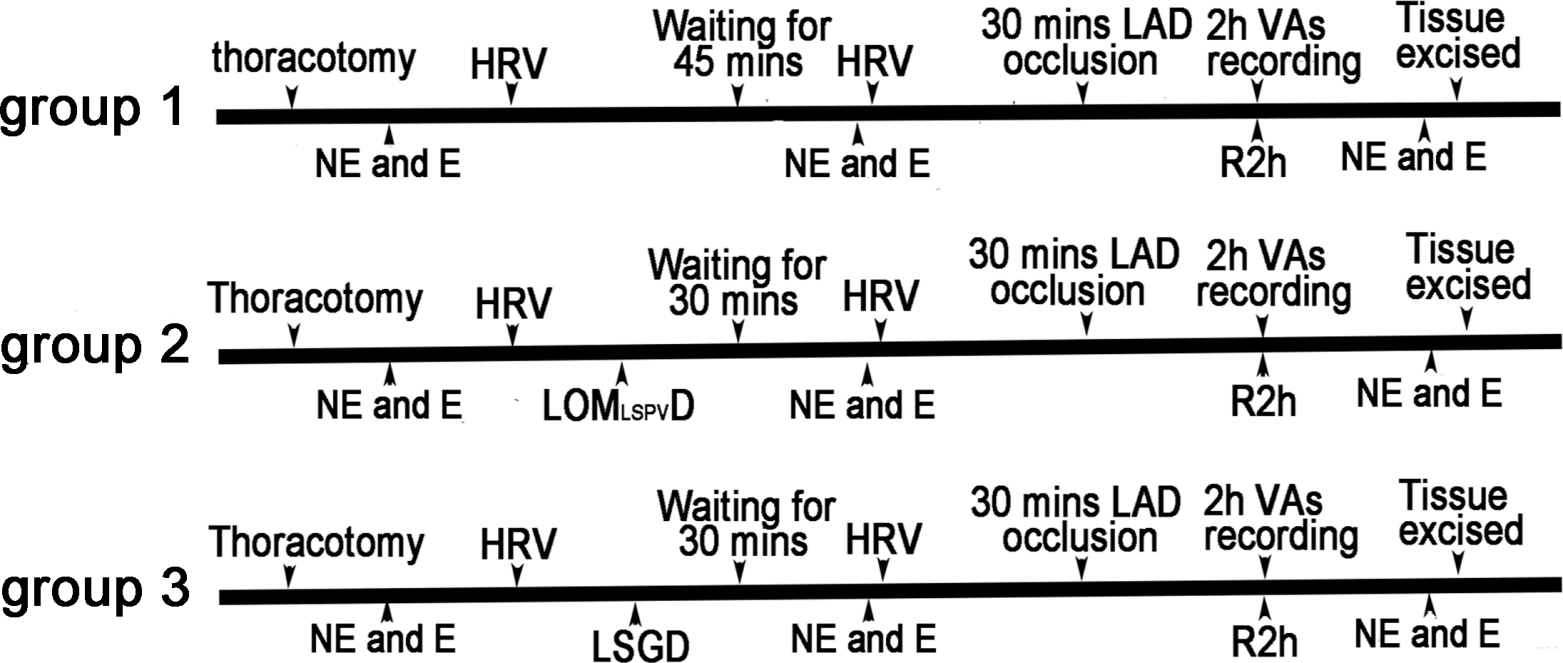
Experimental protocols. IR: ischemia and reperfusion; NE: noradrenaline; E: epinephrine; HRV: heart rate variability; LSGD: left stellate ganglion denervation; LOM_LSPV_D: LOM_LSPV_ denervation; LAD: left anterior descending coronary artery; Vas: ventricular arrhythmias; R2 h: reperfusion for 2 h.

All experimental protocols presented in this study conformed to the Guidelines for the Care and Use of Laboratory Animals published by the US National Institutes of Health (NIH Publication, revised 1996) and were approved by the Renmin Hospital of Wuhan University. Thirty-three male hybrid canines (15–20 kg) were randomly divided into the following three groups, as shown in Fig 1:

Group 1, ischemia and reperfusion (IR) (n = 11): after the canines were anesthetized, they underwent surgical manipulations in which the left anterior ascending coronary artery (LAD) was occluded for 30 mins followed by reperfusion for 2 h;

Group 2, LOM_LSPV_ Denervation + IR (n = 9): after the canines were anesthetized, they underwent surgical manipulations to perform LOM_LSPV_ ablation. At 30 mins later, the LAD was occluded for another 30 mins followed by reperfusion for 2 h;

Group 3, LSG Denervation + IR (n = 13): after the canines were anesthetized, they underwent surgical manipulations to perform LSG ablation. At 30 mins later, the LAD was occluded for another 30 mins followed by reperfusion for 2 h.

The canines were anesthetized with 3.0% sodium pentobarbital (30 mg/kg, iv) followed by maintenance doses (5 mg/kg/h).

A LEAD Electrophysiology Management System (Lead 7000 series, jinjiang, Chengdu, China) was used to record blood pressure, electrocardiograms (high-pass filter: 0.05 Hz, low-pass filter: 20 Hz) and LOM potential (high-pass filter: 60 Hz, low-pass filter: 600 Hz). A Cardiac Ablation Generator (HL-100F jinjiang, Chengdu, China) was used to ablate LOM_LSPV_. An electronic stimulator (Grass-S88, Astro-Med, West Warwick, Rhode Island) was used to stimulate LOM_LSPV_.

Post-thoracotomy, the left anterior ascending coronary artery was blunt separated with the use of hemostatic forceps and with minimal damage to the surrounding tissues. Then the left anterior ascending coronary artery was occluded using a 3–0 suture with tubing interposed between the artery and suture at the level at which it sent out the first diagonal branch. At 30 mins later, the suture was cut, and the tissue was reperfused for 2 h.

A 4 ml volume of blood was collected from the jugular vein of each canine in the baseline (BS) state after thoracotomy, at 30 mins after LOM_LSPV_ Denervation or LSG Denervation and after 2 h of reperfusion. These blood samples were set aside for 10 mins and then centrifuged (3000 r, 15 mins). Only the serum was collected, and it was then frozen at -80°C to detect NE and E.

### The LOM_LSPV_ denervation

**Fig 2:**
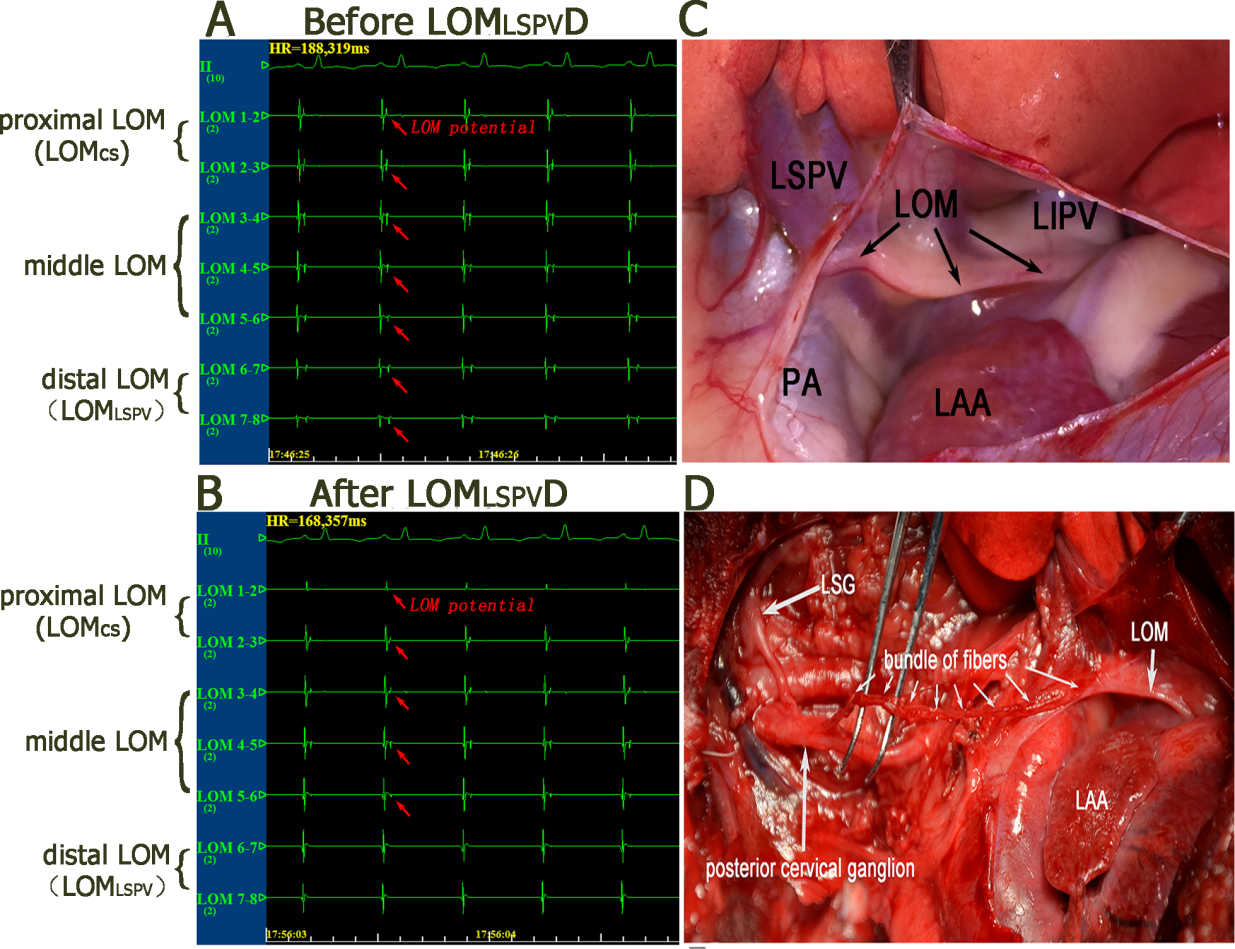
The potential and anatomy position of LOM. (A) The potential of LOM from its proximal to its distal portion. (B) The potential of the distal portion of the LOM dismissed. (C) The LOM originates from the coronary sinus and ends in the pericardium near the LSPV. (D) The LSG sends out a cluster of fibers that end in the posterior cervical ganglion, which sends out a bundle of fibers that innervate the distal part of the LOM. LOM: the ligament of Marshall; LSPV, LIPV: left superior, left inferior pulmonary vein; PA: pulmonary artery; LAA: left atrial appendage.

The LOM originates from the coronary sinus and ends in the pericardium near the LSPV, as shown in Fig 2C. LOM_LSPV_ denervation was performed as previously described [9]. Briefly, the potential of the LOM was recorded with an octupole catheter (the electrode width was 2 mm, and the gap between two adjacent electrodes was 2 mm), as show in Fig 2A. Then, the distal portion of the LOM (LOM_LSPV_) was stimulated with high-frequency (HF) stimulation (20Hz, 0.1 ms duration, square waves) via a stimulator. The LOM_LSPV_ was ablated with an irrigated large-tip (3.5 mm) electrode catheter (Biosense Webster Inc., Diamond Bar, CA, USA) by delivering a radiofrequency current (35 W, 45°C), with minimal damage to the underlying atrial tissue until the LOM_LSPV_ potential was eliminated and the blood pressure didn’t increase (data was not shown), as show in Fig 2B.

### LSG denervation

A left thoracotomy was performed at the second intercostal space. The caudal half of the LSG was identified as the increase in blood pressure that occurred in response to an electrical stimulation (2 ms pulse width, 20 s, 15 mA) that was applied via an irrigated large-tip (3.5 mm) electrode catheter (Biosense Webster Inc., Diamond Bar, CA, USA). The caudal half of the LSG was ablated with the same electrode catheter by delivering a radiofrequency current (35 W, 45°C). To confirm the adequacy of ablation, at 5 min after each ablation, the caudal half of the LSG was electrically stimulated (2 ms pulse width, 20 s, 15 mA). The ablation was considered successful when electrical stimulation induced no changes in blood pressure.

### Ventricular arrhythmia recording

Electrocardiograms were continuously recorded. The numbers of ventricular premature beat (VPB), salvo of VPB, ventricular tachycardia (VT) (defined as three or more consecutive VPB), VT duration (VTD) and ventricular fibrillation (VF) (defined as tachycardia with a random ECG morphology and the loss of arterial blood pressure) were counted.

### Hematoxylin-Eosin (HE) and immunohistochemistry staining

Two LOM_LSPV_ were quickly excised after reperfusion from animals in group 1 and group 3. They were then fixed in 4% paraformaldehyde for 48 h, dehydrated, embedded in paraffin and cut into 4 μm-thick sections. Some sections were stained with HE for tissue diagnosis.

Vagus nerves which lay within the adipose tissue on the surface of the pulmonary artery were collected for CHaT positive control staining.

Tyrosine hydroxylase (TH) is the rate-limiting enzyme in the biosynthesis of catecholamine neurotransmitters (including L-dopamine, epinephrine and norepinephrine). Acetylcholine transferase (CHAT) is a marker enzyme for cholinergic neurons. TH staining and CHAT staining were used to identify the sympathetic nerves and parasympathetic ones. Briefly, after the sections were mounted and dewaxed, antigen repair was performed with a microwave, endoperoxidase activity was blocked by 3% H2O2, and the sections were blocked in rabbit serum. The sections were then probed with primary antibodies, including anti-tyrosine hydroxylase (TH) (diluted 1:1000, Servicebio, Wuhan, China) and anti-choline acetyltransferase (CHAT) (diluted 1:500, Servicebio, Wuhan, China), overnight at 4°C. After the sections were incubated with the appropriate secondary antibodies, the sections were visualized with 3,3’-diaminobenzidine substrate and then stained with hematoxylin to visualize cell nuclei. Stained samples were photographed under a microscope at 400× amplification.

### Heart rate variability (HRV)

To evaluate sympathetic nervous activity, HRV was measured. LabChart (ADInstruments, shanghai, China) was used in a standard II configuration to record electrocardiograph for 30 min at a BS after thoracotomy and for 30 mins after LOM_LSPV_ ablation or LSG ablation. The following frequency parameters were assessed: Low frequency (LF) (0.040–0.015 Hz), high frequency (HF) (0.15–0.5 Hz) and the ratio of LF to HF (LF/HF). LF is a quantitative marker for cardiac sympathetic tone; HF is controlled by the parasympathetic nerves, presumably in the SA node, and the LF/HF ratio reflected the balance between sympathetic tone and parasympathetic tone [7,10].

### Noradrenaline (NE) and epinephrine (E) levels

To evaluate sympathetic activity, the serum expression levels of NE and E were determined with a commercial ELISA kit (Elabscience Biotechnology Co., Ltd, Wuhan, China) in strict accordance with the manufacturer’s instructions. The detectable range of concentrations was 0.313–20 ng/ml or NE and 31.25–2000 pg/ml for E.

### Statistical analysis

SPSS 20.0 was used for all statistical analyses. Quantitative data are expressed as means ± SD or medians ± quartiles. Enumeration data are expressed as fractions. The Kolmogorov-Smirnov test was used to check for a normal distribution. One-way ANOVA was used to analyze comparisons among groups, and Scheffe’s post-hoc test or Tamhane’s T2 test was used for multiple comparisons. Paired t tests were used for comparisons of HRV. Fisher’s exact test was used for enumeration data comparisons. The Kruskal-Wallis test was used for comparisons among groups with non-normal distribution data, and pairwise post-hoc comparisons were used for multiple comparisons. A P value < 0.05 was considered statistically significant.

## Results

### LOM_LSPV_ staining

**Fig 3:**
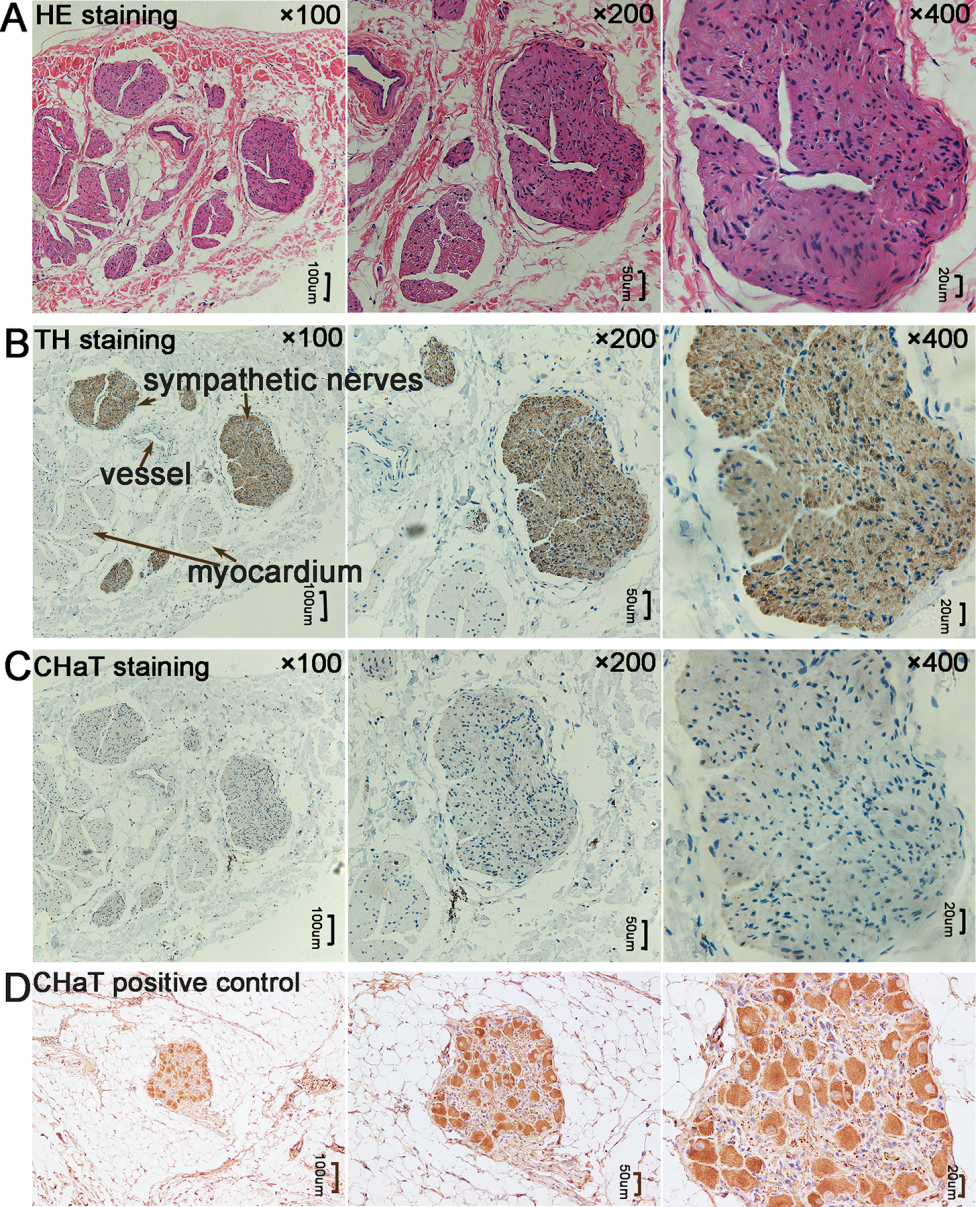
The LOM_LSPV_ contains abundant sympathetic but not parasympathetic nerves. (A) HE staining; (B) positive TH staining; (C) negative CHAT staining; (D) CHAT positive control.

Samples obtained from the LOM_LSPV_ of animals in group 1 and group 3 contained bundles of fibers (Fig 3A) that consisted of an abundance of sympathetic (Fig 3B) but not parasympathetic (Fig 3C) nerves.

### HRV in the baseline state after thoracotomy and 30 mins after LOM_LSPV_ denervation or LSG denervation in the three groups

After a 45-min blanking period, sympathetic nervous activity (LF: 4.20 ± 3.40 vs 4.28 ± 3.36 ms^2^; LF/HF: 1.62 ± 1.19 vs 1.93 ± 1.83) and parasympathetic nervous activity (HF: 3.21 ± 2.54 ms^2^ vs 3.13 ± 2.57 ms^2^) were similar to those observed at BS in group 1. Both LOM_LSPV_ denervation and LSG Denervation significantly reduce cardiac sympathetic nervous activity (lower LF: 1.05±1.14 vs 2.36 ± 2.34 ms^2^ in group 2 and 0.51 ± 0.39 vs 2.24 ± 1.53 ms^2^ in group 3; and LF/HF ratio: 0.35±0.21 vs 1.65 ± 1.04 in group 2 and 0.27±0.28 vs 1.42 ± 0.77 in group 3; all P<0.05) but neither had a significant effect on parasympathetic nervous activity though which showed an increasing trend (HF: 2.69±2.00 vs 2.01 ± 2.14 in group 2 and 2.66±1.74 vs 1.95 ± 1.60 in group 3, P>0.05).

### Concentrations of NE and E in a baseline state after thoracotomy, at 30 mins after LOM_LSPV_ or LSG ablation, and after 2 h of reperfusion in the three groups

**Fig 4:**
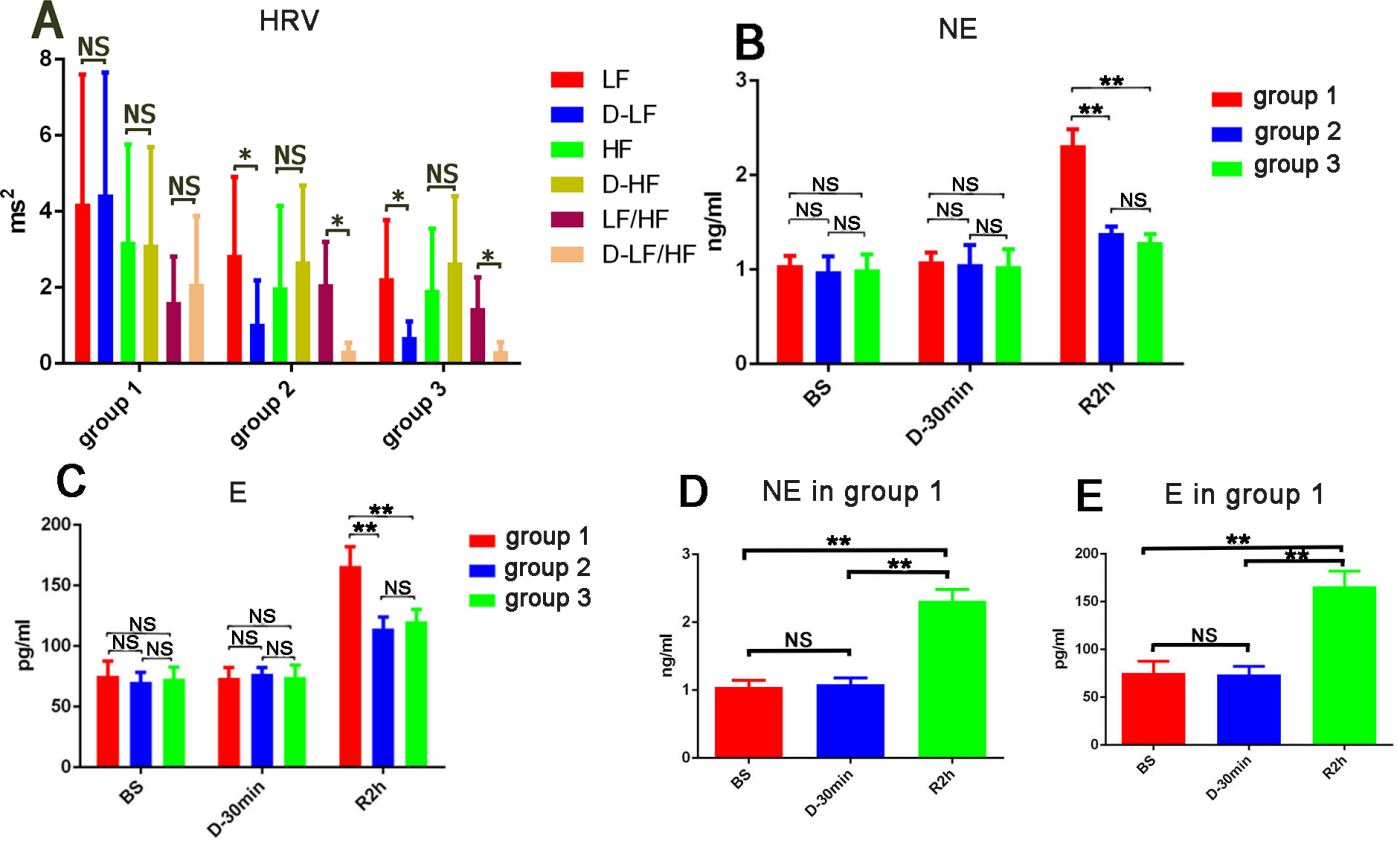
Both LOM_LSPV_D and LSGD reduced the cardiac sympathetic tone (LF, LF/HF and iNE) but not parasympathetic tone (HF). (A) LOM_LSPV_D and LSGD exhibit reduced LF and LF/HF ratios but exhibit no change in HF in BS. (B) LOM_LSPV_D and LSGD exhibit reduced serum E concentrations after 2 h of reperfusion. (C) LOM_LSPV_D and LSGD exhibit reduced serum NE concentrations after 2 h of reperfusion (as above). (D) After 2 h of reperfusion, serum NE concentrations were significantly higher in group 1. (E) After 2 h of reperfusion, serum E concentrations were significantly higher in group 1 (n = 7). BS: baseline state. D-30 min: 45 min after the pericardium was cut open in group 1 or 30 min after sympathetic nerve denervation, R2 h: 2 h after reperfusion, LF: low frequency, HF: high frequency; D-LF, D-HF and D-LF/HF: the values for LF, HF and the LF/HF ratio at 45 min after the pericardium was cut open in group 1 or 30 min after sympathetic nerve denervation in group 2 and group 3; * P<0.05, ** P<0.001. Group 1: n = 11 before reperfusion, n = 7 after reperfusion; Group 2: n = 9 before reperfusion, n = 7 after reperfusion; Group 3: n = 13 before reperfusion, n = 12 after reperfusion.

In BS, the three groups had the similar serum levels of NE (1.05±0.10 ng/ml VS 0.98±0.16 ng/ml VS 1.00±0.14 ng/ml in groups 1, 2, and 3, respectively; P>0.05) and E (75.49±12.27 pg/ml VS 70.60±7.62 pg/ml VS 73.00±9.81 pg/ml, respectively; P>0.05). After a 45-min blanking period in group 1, at 30 min after LOM_LSPV_ denervation in group 2, or at 30 min after LSG Denervation in group 3, the three groups had similar serum levels of NE (1.09±0.10 ng/ml VS 1.06±0.20 ng/ml VS 1.04±0.18 ng/ml, respectively; P>0.05) and E (73.75±8.62 pg/ml vs 77.10±5.09 pg/ml vs 74.60±9.81 pg/ml, respectively; P>0.05). LOM_LSPV_ denervation and LSG Denervation did not result in a lower than basal level of expression of NE (0.98±0.16 ng/ml VS 1.06±0.20 ng/ml and 1.00±0.14 ng/ml vs 1.04±0.18 ng/ml, respectively; P>0.05) or E (70.60±7.62 pg/ml vs 77.10±5.09 pg/ml and 73.00±9.81 pg/ml vs 74.60±9.81 pg/ml, respectively; P>0.05). After 2 h of reperfusion, NE levels were lower in groups 2 (1.39±0.068 ng/ml) and 3 (1.29±0.081 ng/ml) than in group 1 (2.32±0.17 ng/ml) (P<0.05), as were E levels (groups 1, 2, and 3; 166.18±15.78 pg/ml, 114.64±9.22 pg/ml, and 112.60±9.69 pg/ml, respectively; P<0.05). The levels of NE and E were similar between groups 2 and 3 (all P>0.05) (Fig 4B and 4C).

In the IR group, after a blanking period of 45 min, NE and E concentrations were similar to those obtained in a BS. However, the expression levels of NE and E were significantly higher after 2 h of reperfusion (both P<0.05) (Fig 4D and 4E).

### LOM_LSPV_ denervation and LSG denervation prevent VAs

**Fig 5:**
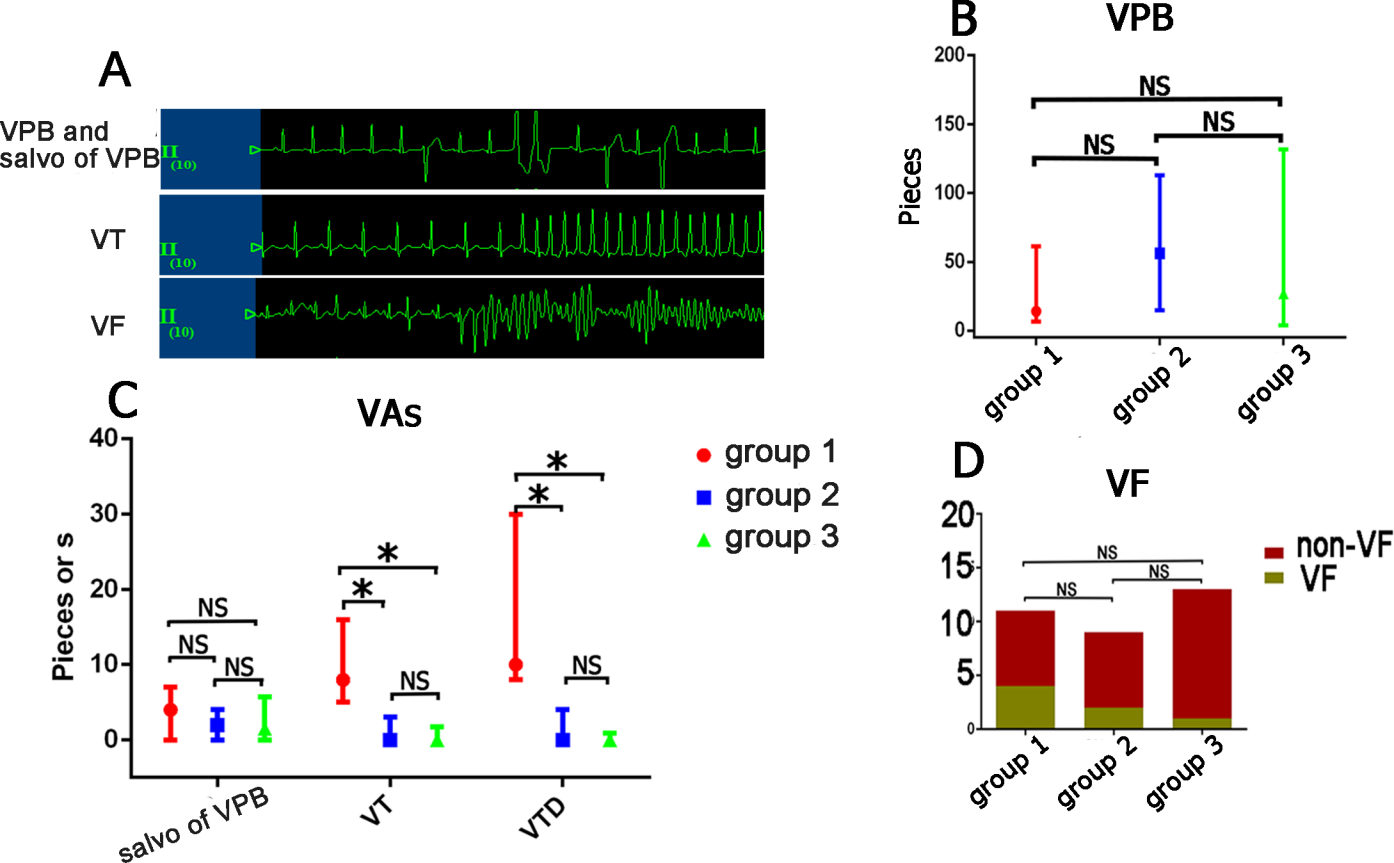
LOM_LSPV_D and LSGD similarly suppressed the prevalence of VT and VTD. (A) VPB, Salvo of VPB, VT and VF recording segments; (B) LOM_LSPV_ denervation and LSG denervation similarly reduced the prevalences of VT and VTD but not VPB or Salvo of VPB; (C) VF showed a decreasing trend in the group 2 and Group 3; VPB: ventricular premature beat, Salvo of VPB: Salvo ventricular premature beat, VT: ventricular tachycardia; VTD: the duration of VT, VF: ventricular fibrillation. * P<0.05, ** P<0.001. Group 1: n = 7; Group 2: n = 7; Group 3: n = 12.

In group 1, VAs, including VPB, salvo of VPB and VT, frequently occurred during the 2 h reperfusion period (Fig 5A). No atrial fibrillation or atrial flutter was observed. All types of VA occurred mainly during the first 30 mins after reperfusion, and the vast majority of VFs (5/7) occurred during the beginning of the reperfusion period. The prevalence of VT was lower (0±3.00 in group 2, 0±1.75 in group 3 vs 8.00±11.00 in group 1, P<0.05) and VTD was shorter VTD (0 ± 4 s, 0±0.88s vs 10.0 ± 22.00s, P<0.05) in groups 2 and 3 than in group 1, but the prevalences of VPB (26.00 ± 95.00, 27.00 ± 127.75 vs 13.00 ± 55.00) and salvo of VPB (2.00 ± 4.00, 1.50 ± 5.75 vs 4.00 ± 7.00) were not significantly altered (Fig 5B and 5C). Although the incidence of VF was not significantly lower in groups 2 and 3 (2/9, 1/13 vs 4/11, P = 0.268), it did show a decreasing trend (Fig 5D). The prevalences of VPB, salvo of VPB, VT, VF and VTD were similar between groups 2 and 3 (all P>0.05)

## Discussion

### Main findings

In the present study, we found that 1) LOM_LSPV_ contained bundles of sympathetic but not parasympathetic nerves. 2) LOM_LSPV_ denervation and LSG Denervation similarly decreased sympathetic nerve activity (LF and LF/HF), but not parasympathetic nerve activity (HF), 3) Both LOM_LSPV_ denervation and LSG denervation reduced the prevalence and duration of VT. These data indicate that LOM_LSPV_ denervation was a sufficient cardiac sympathetic denervation approach. LOM_LSPV_ denervation could reduce the incidence of reperfusion-induced VAs and these effects were comparable to those of LSG devervation.

### The LSG, the SNS and IR-related VAs

A causal link has been established between sympathetic nerve over-activation and VAs. Support for the notion that LSG activity is a hallmark of cardiac sympathetic nerve tone has been demonstrated in both animal models and clinical trials. Zhou et al. [11] found that VAs were accompanied by an earlier increase in LSG activity, and LSG denervation suppressed cardiac events in patients with long QT syndrome, catecholaminergic polymorphic ventricular tachycardia, myocardial infarction and refractory arrhythmia [12–14]. In the present study, we further demonstrate that LSG denervation markedly reduced the prevalences of VT and VTD in a canine myocardial IR model by suppressing cardiac sympathetic nervous activity (LF/HF, NE and E).

### LOM_LSPV_, SNS and IR-related VAs

The LOM, a structure of the epicardium that contains vessels and bundles of fibers and nerves and atrial myocardium, is an important segment of the nerve circuit between the LSG and cardiac sympathetic innervation [15]. Both a previous [16] and the present study found that a cluster of fibers sent by the LSG entered the posterior cervical ganglion, which sent a bundle of fibers that innervated the LOM, as show in Fig 2D. As for its functions, in our previous study [9], we demonstrated that LOM_LSPV_ ablation sufficiently inhibited the blood pressure-increasing effects of LSG stimulation in addition to lowering the prevalence of VT/VF in a Cesium-induced long QT model. As for its histology, previous studies [6] have demonstrated that autonomic nerves are unevenly distributed in the LOM, with the LOM_CS_ containing abundant parasympathetic nerves and the LOM_LSPV_ containing abundant sympathetic nerves. The results of our study further confirm that LOM_LSPV_ is rich in sympathetic but not parasympathetic nerves, as show in Fig 3. These findings collectively suggest that LOM_LSPV_ plays an important role in cardiac sympathetic circuits. In the present study, most importantly, we found that the LOM_LSPV_ denervation significantly suppressed cardiac sympathetic tone and reduced the prevalence of VT and VTD in myocardial IR. Owing to the small sample size, the incidence of VF (4/11 in group 1, 2/9 in group 2, and 1/13 in group 3) was not significantly reduced in groups 2 and 3, although both groups showed a decreasing trend. All of these findings suggest that LOM_LSPV_ denervation can reduce VA severity and suppress the prevalence of malignant VAs by suppressing sympathetic nervous tone.

### LOM_LSPV_ denervation and LSG denervation

In the present study, we found that both LOM_LSPV_ denervation and LSG denervation sufficiently suppressed sympathetic nervous tone and reduced the prevalence of malignant VAs, indicating that LOM_LSPV_ denervation is an efficient method for cardiac sympathetic denervation.

LOM_LSPV_ denervation and LSG denervation had no effects on the systemic concentrations of NE and E in the BS but significantly reduced LF values and the ratio of LF/HF, indicating that LOM_LSPV_ denervation and LSG specifically reduced cardiac sympathetic tone. However, LOM_LSPV_ denervation and LSG denervation sufficiently inhibited the increases in the expression of NE and E induced by IR. NE and E concentrations and HRV results suggested that LOM_LSPV_ denervation and LSG denervation can sufficiently inhibit the dramatic increase in sympathetic activity induced by myocardial IR.

## Clinical application

Reperfusion therapies are the most effective therapies for acute myocardial infarcts. However, the incidence of reperfusion arrhythmias, including lethal VAs, such as VT and VF, is as high as 50%-80%. Cardiac sympathetic denervation is a sufficiently approach that acts by suppressing the incidence of VAs. Surgical LSG denervation can be used to specifically ablate the lower one-third of the LSG, but also involves subjecting the patient to a grave wound. Percutaneous LSG denervation is non-specific and always results in damage other branches of the LSG that innervate the head, neck and diaphragm, causing undesired complications, such as Horner’s syndrome, seriously reducing the quality of life of the patient. In the present study, we demonstrate that LOM_LSPV_ denervation is an effective method for performing cardiac sympathetic denervation because it selectively targets the cardiac SNS and avoids extracardiac complications. It seems feasible that LOM_LSPV_ denervation could be achieved via the infusion of anhydrous alcohol into the distal LOM with a balloon during a percutaneous coronary intervention procedure. LOM_LSPV_ denervation could potentially become an approach to reducing VAs in patients with severe RVAs.

## Conclusion

In conclusion, LOM_LSPV_ denervation may reduce the prevalence of VT by suppressing SNS activity, and its effects were comparable to those achieved by LSG denervation. The anti-arrhythmic effects of LOM_LSPV_ denervation may be related to the inhibition of the expression of NE, E in myocardial IR.

## Limitations

The present study has two main limitations. First, because the sample size was small, there was no significant difference in the incidence of VF among the three groups, although there was a decreasing trend in groups 2 and 3. Second, anesthesia affect the activity of autonomic nerves. Hence, although we anesthetized the canines using the same standards (30 mg/kg iv), the canines’ individual tolerances and differences in operating times during the experiment might have affected the results.

## Supporting information

S1 Data Minimal data set. The original data of the expression of NE and E, the prevalence of VAs and the value of HRV were included in this file. NE: noradrenaline; E: epinephrine; VAs: ventricular arrhythmias; HRV: heart rate variability.
(XLSX)
